# Lactate on emergency department arrival as a predictor of in-hospital mortality in necrotizing fasciitis: a retrospective study

**DOI:** 10.1186/s13018-019-1108-y

**Published:** 2019-03-06

**Authors:** Chia-Peng Chang, Wen-Chih Fann, Shu-Ruei Wu, Chun-Nan Lin, Cheng-Ting Hsiao

**Affiliations:** 1 Department of Emergency Medicine, Chang Gung Memorial Hospital, No.6, Sec. W., Jiapu Rd., Puzi City, Chiayi County 613 Taiwan, Republic of China; 2 Department of Medicine, Chang Gung University, Taoyuan, Taiwan; 3 Department of Pediatrics, Kaohsiung Veterans General Hospital, Kaohsiung, Taiwan

**Keywords:** Lactate, Necrotizing fasciitis, In-hospital mortality, Emergency department, SOFA score

## Abstract

**Background:**

Hyperlactatemia is known to be associated with adverse outcome in critical illness. In this study, we attempted to identify if hyperlactatemia on emergency department (ED) arrival is a reliable predictor for in-hospital mortality in necrotizing fasciitis (NF) patients.

**Method:**

A prospective cohort study of hospitalized patients with NF was conducted in two tertiary teaching hospitals in Taiwan between March 2010 and March 2018. Blood samples were collected in the ED upon arrival, and the lactate levels were determined. Sequential organ failure assessment (SOFA) scores were calculated during the first 24 h after admission. All collected data were statistically analyzed.

**Result:**

Of the 707 NF patients, 40 (5.66%) died in the hospital. The median (interquartile range) blood lactate level in all NF patients was 3.6 mmol/l (2.2–4.8). The blood lactate level upon ED arrival was significantly associated with mortality (odds ratio [OR] = 1.35; 95% confidence interval [CI], 1.30–1.46; *P* < 0.001), even after adjustment for age and SOFA score (OR = 1.27; *P* < 0.001). Multivariate regression analysis showed that a high blood lactate level (OR = 1.17; 95% CI, 1.07–1.29; *P* = 0.001) and a high SOFA score (OR = 1.15; 95% CI, 1.11–1.20; *P* < 0.001) were independent risk factors for in-hospital mortality in NF. Blood lactate achieved an area under-the-receiver-operating-characteristic curve (AUC) of 0.79 (*P* < 0.001) for predicting mortality that was similar to that of SOFA score (AUC = 0.82; *P* < 0.001). Blood lactate displayed a sensitivity of 62% and a specificity of 86% in predicting mortality at the optimal cutoff value of 5.80 mmol/l.

**Conclusion:**

In necrotizing fasciitis patients, hyperlactatemia on ED arrival is independently associated with in-hospital mortality. NF patients with hyperlactatemia on ED arrival should be closely monitored for signs of deterioration and consider early and aggressive intervention to prevent mortality.

## Introduction

Necrotizing fasciitis (NF) is a serious form of infection involving rapidly spreading inflammation and extensive necrosis of the skin, subcutaneous tissue, and superficial fascia [1]. The treatment of choice for NF is rapid surgical debridement and broad-spectrum antibiotic therapy [2]. Even with aggressive treatment, patients may suffer mortality and significant morbidity such as amputation and organ failure [3–5].

Lactate is generated by the skeletal muscle, erythrocytes, the brain, and the gut. In a normal setting, aerobic metabolism converts pyruvate through the citric acid cycle to generate 38 molecules of ATP. This process is facilitated by the enzyme pyruvate dehydrogenase, an ample supply of oxygen and substrate (glucose), and functioning mitochondria. In relative oxygen-deficient states, anaerobic metabolism fails to convert pyruvate to acetyl-CoA through the citric acid cycle. Instead, two molecules of ATP and two molecules of lactate are generated by the conversion of pyruvate to lactate using the enzyme lactate dehydrogenase [6]. In healthy individuals, there is a continuous cycle of lactate production and metabolism, which ensures that blood lactate concentrations are normally low. Higher blood lactate concentrations occur when lactate production exceeds clearance, when clearance capacity is decreased or more frequently when both occur simultaneously [7]. Elevated lactate was shown to be independently associated with mortality rate in critically ill patients [8]. Even intermediate initial serum lactate was an indicator of mortality of organ dysfunction and shock in severe sepsis patients in the emergency department (ED) [9]. In hospitalized patients, increased lactate indicated high mortality, mechanical ventilation, vasopressor requirement, and a high incidence of ICU admission [10–12]. Lactate performed well in risk assessment studies in septic patients; the cutoff values (2 and 4 mmol/l) were quite similar in different reports [11, 13, 14]. A Denmark study indicated the association between lactate level and mortality varied across different diagnostic groups. Lactate level showed to be useful in patients with infection, trauma, cardiac disease, and gastrointestinal diseases [15]. However, there was limited data about the prognostic value of lactate among infectious diseases. Schwartz et.al reported in a retrospective study of 174 patients that admission hyperlactatemia was an independent predictor of increased limb loss and mortality in NF [16]. The clinical significance of serum lactate was established over the last decade. Trends in lactate concentration over time reflect the clinical response of patients to resuscitation and surgical intervention. However, lactate level is not routinely monitored at the time of patient arrival as standard of care. Emergency department (ED) physicians are concerned with the resuscitation and identification risk of NF patients. Early recognition of NF patients who are at high risk for mortality allows for timely changes in therapy and improves the overall outcomes. We try to measure the association of the initial serum lactate level in ED with in-hospital mortality of NF. Our objective in this study was to retrospectively evaluate whether hyperlactatemia in ED is associated with increased in-hospital mortality in NF patients.

## Material and methods

### Patient selection

The institutional review board of two hospitals approved this prospective study. In all, 707 patients were enrolled based on two criteria, (1) surgically proven diagnosis of NF and (2) treatment received between March 2010 and March 2018 in our hospital. All patients were assessed by emergency physicians as soon as they were admitted. They received broad-spectrum antibiotic treatment for anaerobic and aerobic bacteria as well as early surgical debridement including fasciotomy or primary amputation post-diagnosis. Each patient’s medical record was screened for documentation of NF to confirm the diagnosis. Blood samples were collected in the ED upon arrival, and the lactate levels were determined. SOFA scores were calculated during the first 24 h after admission. Baseline demographic characteristics, laboratory findings, and serum lactate were compared between survivor and non-survivor groups.

### Blood sample collection and measurement

To determine the blood lactate level at ED arrival, a peripheral venous sample was collected in the first hour upon patient arrival to ED. The sample was collected directly into a tube containing anticoagulant. The blood concentration of lactate was measured immediately using an automatic biomedical blood gas analyzer (CCX, NOVA Biomedical, USA) as part of a routine panel of blood gas tests in our clinical laboratory with the electrode method, and it was expressed in millimoles per liter (mmol/l). The coefficient of variation was 7.5% at the low level and 5.0% at the high level. The normal range is 0.5–2 mmol/l. The laboratory investigators were blinded to the sample sources and clinical outcomes.

### SOFA score

Sequential organ failure assessment (SOFA) is one of the scoring systems used for assessing the severity of disease in critically ill patients and predicting their outcome [17, 18]. This system was introduced in 1996 and it performs based on evaluating the function of six vital organs of respiratory, coagulation, cardiovascular and circulatory, liver, central nervous system, and renal. This tool is easy to use and evaluates the status of the mentioned organs systematically and continuously during hospitalization [19]. Studies have shown that SOFA score is able to provide valuable prognostic data regarding in-hospital mortality of septic patients [17, 20].

### Data analysis

We reviewed charts and recorded variables including age, systolic and diastolic blood pressure at triage, comorbidities, discharge diagnosis, and mortality or survival on discharge. Patients with in-hospital mortality were defined as a death occurring in the hospital after admission, also known as “non-survivor group,” otherwise as “survivor group.” We defined these variables as follows: episodes of hypotension, episodes of systolic blood pressure less than 90 mmHg at the ED; hypothermia, body temperature less than 36 °C at the ED; hyperthermia, body temperature ≥ 38 °C at the ED; acidosis, pH less than 7.35 in arterial blood gas test at the ED; coagulopathy, a prolonged prothrombin time test (international normalized ratio) result greater than 1.5 at the ED; thrombocytopenia, platelet counts less than 100 × 10^3^ μL at the ED; anemia, hemoglobin less than 10 mg/dL at the ED; and episodes of SpO2 < 90%, oxygen saturation less than 90% at the ED. Prothrombin time test, hemoglobin, platelet counts, blood gas test, serum lactate, albumin, sodium, creatinine, and CRP (C-reactive protein) were assessed by first laboratory analyses in ED.

Statistical analyses were done by using SPSS 20.0. Assumptions of normality and homogeneity of variance were first checked. For continuous variables with a skewed distribution, descriptive results were expressed as medians and interquartile ranges. The Mann–Whitney *U* test was used to determine the differences between the two groups, and the Kruskal-Wallis *H* test was used to analyze the differences among groups. Univariate binary and multivariate logistic regression analyses were performed to investigate whether blood lactate was independently associated with in-hospital mortality. Analysis of the area under the curve (AUC) of the Receiver Operating Characteristic (ROC) curve was constructed to assess the predictive strength. Sensitivity, specificity, and positive and negative likelihood ratios and predictive values were calculated at different cutoff values. Optimal cutoff points to maximize both sensitivity and specificity were also determined. Differences with *P* values < 0.05 were considered to be statistically significant.

## Results

### Patient characteristics

Of the total 707 NF patients, 40 (5.66%) died in the hospital. Patients who were discharged to home were considered to have a favorable outcome. The demographic and clinical characteristics and laboratory findings on the ED arrival are compared between survivors and non-survivors in Table 1. The concentration of blood lactate upon ED arrival in non-survivors was significantly higher than in survivors (*P* < 0.001). SOFA scores in non-survivors were also significantly higher than those in survivors, which were calculated during the first 24 h after admission. In the analysis of initial variables recorded at ED, there were statistically significant differences with serum albumin (3.1 vs. 2.6 g/dl, *P* < 0.001), serum creatinine (1.7 vs. 2.4 mg/dl, *P* < 0.01), and CRP level (124.1 vs. 161.7 mg/dl, *P* < 0.01).

**Table 1:** Comparison of demographic and clinical characteristics and laboratory findings on ED arrival between survival and non-survival NF patients

Characteristics	Survivors (*n* = 667)	Non-survivors (*n* = 40)	*P* value
Age, years, mean	57.2 (35.7–69.8)	60.7 (39.3–82.6)	0.09
SBP at triage, mmHg	146.5 (124.6–189.9)	141.4 (104.6–198.5)	0.93
DBP at triage, mmHg	85.1 (55.6–101.7)	78.7 (44.1–99.5)	079
SOFA score	4 (0–6)	9 (5–23)	< 0.001
Episodes of hypotension, *n* (%)	86 (12.90)	19 (47.50)	< 0.01
Hypothermia (BT < 36), n (%)	78 (11.70)	10 (25.0)	0.56
Hyperthermia (BT ≥ 38), *n* (%)	150 (22.49)	9 (22.50)	0.94
Acidosis, *n* (%)	106 (16.04)	10 (25.0)	< 0.001
Coagulopathy, *n* (%)	95 (14.24)	11 (27.5)	< 0.001
Thrombocytopenia, *n* (%)	69 (10.34)	7 (17.5)	0.23
Anemia, *n* (%)	88 (13.19)	11 (27.50)	0.08
Episode of SpO2 < 90%, *n* (%)	65 (9.75)	6 (15.0)	0.07
Blood lactate (mmol/l)	2.8 (0.5–5.6)	6.6 (1.2–11.8)	< 0.001
Serum albumin (g/dl)	3.1 (2.1–4.8)	2.6 (1.9–3.6)	< 0.001
Serum creatinine (mg/dl)	1.7 (0.5–3.8)	2.4 (0.9–6.6)	< 0.01
Serum glucose (mg/dl)	163 (121.5–188.9)	192 (128.6–246.5)	< 0.01
CRP (mg/dl)	124.1 (56.1–174.5)	161.7 (65.8–205.6)	< 0.01
Diabetes mellitus, *n* (%)	193 (28.94)	14 (35.0)	0.26
Liver cirrhosis, *n* (%)	141 (21.14)	13 (32.5)	0.15
Chronic kidney disease, *n* (%)	207 (31.03)	14 (35.0)	0.34

Values are median [interquartile range]. Numbers in parentheses denote percentages

*CRP* C-reactive protein, *DBP* diastolic blood pressure, *SBP* systolic blood pressure, *SOFA* sequential organ failure assessment

### Comparison of data in NF patients with different concentrations of blood lactate

Blood lactate was detectable with a range of 0.4–19.3 mmol/l in 707 samples. The median blood lactate level measured upon ED arrival was 3.6 mmol/l. Among the NF patients, 562 (79.5%) had a lactate concentration > 2.0 mmol/l., including 275 (38.9%), 141 (19.9%), 84 (11.9%), and 62 (8.8%) who had lactate concentrations of 2.1–4.0, 4.1–6.0, 6.1–8.0, and greater than 8.0 mmol/l, respectively. A comparison of the demographic, clinical characteristics and the laboratory findings collected upon ED arrival and SOFA score among NF patients with different concentrations of serum lactate is shown in Table 2. The incidence of in-hospital mortality was significantly associated with increased blood lactate levels (*P* < 0.001). A significant increase in the SOFA scores (*P* < 0.001), incidence of acidosis, coagulopathy, episodes of hypotension, and the serum creatinine, glucose, and CRP concentrations were associated with increases in the blood lactate levels. In contrast, the serum albumin concentrations (*P* < 0.001) were significantly decreased with increases in the blood lactate levels.

**Table 2:** Comparison of demographic and clinical characteristics and laboratory findings on ED arrival among NF patients with different concentrations of serum lactate

Admission blood lactate, mmol/l	0.0–2.0	2.1–4.0	4.1–6.0	6.1–8.0	> 8.0	*P* value
*n*	145	275	141	84	62	
Age, years	56.1 (30.1–68.9)	57.1 (35.9–71.2)	57.8 (33.8–75.1)	59.3 (41.9–79.2)	60.3 (42.3–85.6)	< 0.001
SBP at triage, mmHg	137.4 (115.8–175.6)	134.5 (115.4–189.7)	130.8 (106.8–174.3)	125.7 (105.2–170.9)	124.8 (103.6–168.7)	0.61
DBP at triage, mmHg	87.6 (51.8–120.5)	85.4 (52.4–117.9)	82.7 (50.7–115.6)	79.5 (42.7–114.2)	76.9 (40.9–108.1)	0.18
Episode of hypotension, *n* (%)	12 (10.3)	30 (10.9)	23 (16.3)	19 (22.6)	21 (33.9)	< 0.001
SOFA score	3 (0–6)	4 (0–6)	5 (1–7)	7 (2–9)	9 (4–16)	< 0.001
Hypothermia (BT < 36), *n* (%)	15 (10.3)	33 (12.0)	18 (12.8)	14 (13.1)	8 (12.5)	0.74
Hyperthermia (BT > =38), *n* (%)	33 (22.8)	70 (25.5)	28 (19.9)	17 (20.2)	11 (17.7)	0.56
Acidosis, *n* (%)	10 (6.9)	34 (12.4)	26 (18.4)	25 (29.8)	21 (33.9)	< 0.001
Coagulopathy, *n* (%)	16 (11.0)	36 (13.1)	22 (15.6)	17 (20.2)	15 (24.2)	< 0.001
Thrombocytopenia, *n* (%)	13 (9.0)	26 (9.5)	11 (7.8)	15 (17.9)	11 (18.4)	0.22
Anemia, *n* (%)	18 (12.4)	37 (13.5)	19 (13.5)	14 (16.7)	11 (18.5)	0.96
Episode of SpO2 < 90%, *n* (%)	11 (7.6)	25 (10.2)	17 (12.1)	11 (13.1)	7 (11.9)	0.77
In-hospital mortality, *n* (%)	3 (2.1)	6 (2.2)	7 (5.0)	11 (13.1)	13 (21.0)	< 0.001
Serum albumin (g/dl)	3.8 (2.6–4.1)	3.7 (2.4–4.6)	3.5 (2.1–3.9)	3.1 (2.1–3.5)	2.8 (1.8–3.4)	< 0.001
Serum creatinine (mg/dl)	1.1 (0.4–2.8)	1.3 (0.5–2.9)	1.7 (0.9–3.8)	2.1 (1.1–5.6)	2.5 (1.4–7.9)	< 0.01
Serum glucose (mg/dl)	128 (94–159)	136 (93–166)	158 (101–196)	169 (105–201)	184 (117–243)	< 0.01
Serum CRP (mg/dl)	98 (15–124)	112 (29–161)	135 (35–184)	169 (51–196)	198 (81–230)	< 0.01
Diabetes mellitus, *n* (%)	38 (26.2)	61 (24.9)	51 (36.1)	31 (36.9)	26 (42.3)	0.06
Liver cirrhosis, *n* (%)	24 (16.6)	45 (18.5)	32 (22.7)	29 (34.5)	24 (38.9)	0.08
Chronic kidney disease, *n* (%)	39 (26.9)	69 (28.2)	50 (35.5)	33 (39.3)	30 (48.7)	< 0.001

Values are median [interquartile range]. Numbers in parentheses denote percentages

*CRP* C-reactive protein, *DBP* diastolic blood pressure, *SBP* systolic blood pressure, *SOFA* sequential organ failure assessment

### Association of blood lactate level with in-hospital mortality

Univariate binary and multivariate logistic regression analyses were performed to investigate whether the blood lactate level upon ED arrival was independently associated with in-hospital mortality (Table 3). Age, SOFA score, laboratory findings collected on the ED arrival, clinical conditions, and comorbidities that were potentially associated with in-hospital mortality were included in the analyses. The following factors were significantly associated with in-hospital mortality in the unadjusted binary logistic regression analysis: SOFA score, acidosis, coagulopathy, episode of hypotension, blood lactate, albumin, creatinine, CRP value, and comorbidity with chronic kidney disease. The odds for in-hospital mortality increased by 35%, for every 1 mmol/l increase in blood lactate (OR = 1.35; 95% CI, 1.30–1.46; *P* < 0.001). The association of blood lactate levels with in-hospital mortality remained significant after adjusting for age and SOFA score (OR = 1.23; 95% CI, 1.19–1.35; *P* < 0.001). Multivariate logistic regression analysis identified blood lactate (OR = 1.17; 95% CI, 1.07–1.29; *P* < 0.001), serum albumin (OR per 1 g/dl increase = 0.86; 95% CI, 0.83–0.89; *P* < 0.001), and SOFA score (OR per 1-point increase = 1.15; 95% CI, 1.11–1.20; *P* < 0.001) as independent factors that were significantly associated with in-hospital mortality in NF. The Hosmer-Lemeshow goodness-of-fit test for the multivariate logistic regression model was not significant (*P* = 0.611), indicating that the model adequately fits the data. Furthermore, the association between blood lactate levels and in-hospital mortality remained significant after adjusting for SOFA score and serum albumin (OR = 1.15; 95% CI, 1.05–1.32; *P* < 0.001).

**Table 3:** Univariate and multivariate logistic regression analyses of variables potentially associated with in-hospital mortality in NF

	Univariate binary logistic regression		Multivariate logistic regression	
	OR (95% CI)	*P* value	OR (95% CI)	*P* value
Age	1.02 (0.96–1.08)	0.542	1.06 (0.96–1.16)	0.249
SOFA score	1.28 (1.06–1.51)	< 0.001	1.15 (1.11–1.20)	< 0.001^e^
Episode of hypotension	1.03 (1.01–1.05)	0.001	1.11 (0.63–1.97)	0.715
Episode of SpO2 < 90%	1.58 (1.07–2.33)	0.325	1.14 (1.02–1.18)	0.459
Acidosis	1.05 (1.01–1.08)	< 0.001	1.03 (0.94–1.12)	0.217
Coagulopathy	1.01 (1.00–1.01)	0.001	1.02 (1.01–1.08)	0.428
Thrombocytopenia	1.03 (0.98–1.08)	0.556	1.01 (0.93–1.03)	0.829
Anemia	1.05 (1.02–1.10)	0.065	0.99 (0.98–1.01)	0.913
Hypothermia	1.01 (1.00–1.01)	0.213	1.03 (0.98–1.07)	0.541
Hyperthermia	1.12 (1.02–1.15)	0.968	1.02 (1.01–1.12)	0.280
Blood lactate	1.35 (1.30–1.46)	< 0.001^a^	1.17 (1.07–1.29)	< 0.001^b, c^
Serum glucose	1.09 (1.01–1.23)	< 0.01	1.94 (0.76–4.75)	0.580
Serum albumin	0.86 (0.83–0.89)	< 0.001^d^	0.92 (0.88–0.96)	< 0.001^d^
Serum CRP	1.11 (1.08–1.13)	< 0.001	1.24 (1.18–3.27)	0.086
Serum creatinine	1.01 (1.00–1.01)	< 0.001	1.12 (0.89–2.35)	0.306
Diabetes mellitus	1.05 (1.02–1.06)	0.133	1.18 (1.02–1.29)	0.102
Liver cirrhosis	1.02 (1.00–1.03)	0.506	1.05 (1.01–1.09)	0.020
Chronic kidney disease	1.02 (1.00–1.02)	< 0.01	1.14 (1.05–1.18)	0.061

The *P* value of the Hosmer-Lemeshow goodness-of-fit test for the multivariate logistic regression model was 0.611

*CI* confidence interval, *CRP* C-reactive protein, *DBP* diastolic blood pressure, *OR* odds ratio, *SBP* systolic blood pressure, *SOFA* sequential organ failure assessment

^a^The association of blood lactate with in-hospital mortality remained significant after adjustment for age and SOFA score (OR = 1.23; 95% CI, 1.19–1.35; *P* < 0.001)

^b^The association of the blood lactate level with in-hospital mortality remained significant after adjustment for serum albumin and SOFA score (OR = 1.15; 95% CI, 1.05–1.32; *P* < 0.001)

^c^Odds ratio represents the increase in risk per 1 mmol/l increase in lactate concentration

^d^Odds ratio per 1 g/dl increase in albumin level

^e^Odds ratio per 1-point increase in SOFA score

### Ability of blood lactate level to predict in-hospital mortality

The predictive ability of blood lactate levels from NF patients upon ED arrival (*n* = 707) for in-hospital mortality (*n* = 40) was assessed (Table 4). The level of blood lactate was predictive of in-hospital mortality and achieved AUC of 0.79 (95% CI, 0.74–0.84; *P* < 0.001). This AUC is similar to SOFA score (AUC = 0.82; 95% CI, 0.78–0.86; *P* < 0.001) for predicting the in-hospital mortality. The *P* value for the comparison of both AUCs was 0.218. Combining blood lactate levels with SOFA score improved the predictive performance (AUC = 0.86; 95% CI 0.83–0.90; *P* < 0.001), which is better than blood lactate alone (*P* = 0.008), but not significantly better than SOFA score alone (*P* = 0.115). The sensitivity and specificity of the blood lactate levels and SOFA scores at the optimal cutoff value to predict in-hospital mortality are shown in Table 4. Blood lactate displayed a sensitivity of 62% and a specificity of 86% at the optimal cutoff value of 5.80 mmol/l. The positive and negative likelihood ratios were 4.5 and 0.45, respectively. SOFA score displayed a sensitivity of 67% and a specificity of 83% for predicting in-hospital mortality at the optimal cutoff score of 7.2, and the positive and negative likelihood ratios were 3.8 and 0.39, respectively. We also calculated the sensitivity and specificity of differing concentrations of blood lactate to predict in-hospital mortality in NF patients (Table 5). At the cutoff value of > 2.0 of 92% and a specificity of 26% for predicting in-hospital mortality, the positive and negative likelihood ratios were 1.3 and 0.23, respectively. The specificity increased to 96%, and the positive likelihood ratio increased to 10.1 at the cutoff value of > 8.0 mmol/l, although the sensitivity decreased to 45%. The OR values of blood lactate at the levels above the set cutoff points are shown in Table 5. In addition, 68.9% of patients had a lactate concentration > 2.5mmol/l in the study, which is suggested to be an optimal cutoff value for lactate to predict deterioration and mortality [21]. Blood lactate displayed a sensitivity of 90% and a specificity of 38% to predict in-hospital mortality at this cutoff value in NF patients.

**Table 4:** Predicting performance of ED blood lactate and SOFA score for in-hospital mortality of NF

	AUC	95% CI	*P* value	Optimal cutoff value	Sensitivity (%)	Specificity (%)
Blood lactate	0.79	0.74–0.84	< 0.001	5.8 mmol/l	62	86
SOFA score	0.82	0.78–0.86	< 0.001	7.2	67	83
Blood lactate combined with SOFA score	0.86	0.83–0.90	< 0.001		64	93

*P* value (comparison of the difference between AUCs)

*P* = 0.218 (between blood lactate and SOFA score)

*P* = 0.115 (between blood lactate combined with SOFA score and SOFA score alone)

*P* = 0.008 (between blood lactate combined with SOFA score and blood lactate alone)

*AUC* area under the receiver-operating-characteristic curve, *CI* confidence interval, *SOFA* sequential organ failure assessment

**Table 5:** Odds ratio, sensitivity, and specificity for ED blood lactate at different concentrations to predict in-hospital mortality of NF

ED lactate, mmol/l	OR^a^ (95% CI)	*P* value	Sensitivity (%)	Specificity (%)	LR+	LR−	PV+	PV−
> 2.0	2.95 (2.27–8.96)	< 0.001	92	26	1.3	0.23	0.55	0.80
> 4.0	4.15 (3.72–9.32)	< 0.001	68	68	2.1	0.41	0.70	0.70
> 6.0	8.02 (6.51–15.27)	< 0.001	57	85	4.9	0.52	0.84	0.66
> 8.0	12.36 (10.54–22.01)	< 0.001	45	96	10.1	0.56	0.91	0.63

*CI* confidence interval, *LR+* likelihood ratio positive, *LR*− likelihood ratio negative, *OR* odds ratio, *PV+* positive predictive value, *PV*− negative predictive value

^a^Odds ratios of blood lactate at the levels above the set cutoff points

## Discussion

This study provides data on blood lactate concentrations and demonstrates that the blood lactate level upon ED arrival is significantly associated with in-hospital mortality in NF patients. A high blood lactate level at ED arrival is predictive of in-hospital mortality in NF patients. Many studies have demonstrated that either admission lactate or peak lactate concentration is associated with mortality in adults [21–24]. To our knowledge, a limited number of studies verified the use of hyperlactatemia as a prognostic index in patients who are admitted via ED [25–28]. Our results are in line with recently published findings that suggest that blood lactate concentration upon ED arrival is predictive of mortality, independent of SOFA score. Patients with contemporaneous blood lactate and SOFA score measurements at admission were enrolled in limited prospective cohort studies [27, 29, 30]. Our study was conducted in a large mixed cohort of NF patients. Blood samples were prospectively collected for assessing the blood lactate concentration. The observation that the extent of absolute hyperlactatemia is strongly linked with mortality independent of illness severity indicates that blood lactate is a useful early predictor in identifying NF patients who are at high risk of death in the ED and hospital. One contribution of this study is the use of SOFA score to control for the severity of the illness. Previous studies suggest that SOFA score is an important tool in predicting mortality and clinical outcomes in critically ill patients [31–34]. The association of ED blood lactate with in-hospital mortality in this study was independent of age and the severity of illness as assessed by SOFA score. The ROC curve analysis in the present study showed that the prognostic accuracy of blood lactate for in-hospital mortality (AUC = 0.79) was similar to that of SOFA score (AUC = 0.82). Because blood lactate at ED and SOFA score obtained within the first 24 h after admission are comparable in predicting mortality, we recommend assessing mortality risk with blood lactate at ED because it is simple to use. Previous studies suggest that there is a confounding relationship between hyperlactatemia and hyperglycemia in nondiabetic critically ill patients [35]. In the present study, there was a significant increase in the blood glucose concentration with an increase in the blood lactate levels, suggesting that hyperglycemia is significantly correlated with hyperlactatemia. We further demonstrated that hyperlactatemia was associated with an increased mortality risk, regardless of the presence of hyperglycemia. This finding suggests that hyperglycemia did not confound the association between the elevated blood lactate level and mortality in this study. In contrast, although hyperglycemia had a significant univariate association with mortality risk, when adjusted for concurrent hyperlactatemia and/or other potential risk factors, hyperglycemia was not a significant predictor of mortality risk in NF patients. The accumulation of lactic acid in the blood is generally associated with metabolic acidosis [36, 37]. This finding raises the question of whether it is possible that the association of hyperlactatemia with mortality could be at least partially attributed to the occurrence of metabolic acidosis. We demonstrated that hyperlactatemia is significantly associated with in-hospital mortality in NF patients, even after adjusting for acidosis. This study was designed to determine the optimal relationship between the sensitivity and specificity of blood lactate assessment in predicting mortality and to set appropriate cutoff values for predicting in-hospital mortality. A systematic review conducted on studies of adult patients suggests that all patients with a lactate at admission above 2.5 mmol/l should be closely monitored for signs of deterioration [21]. In our study, 68.9% of NF patients had a lactate concentration > 2.5 mmol/l. Blood lactate displayed a sensitivity of 90% and a specificity of 38% in predicting in-hospital mortality at 2.5 mmol/l. Specificity is one of the main characteristics of a predictive marker. A test with high sensitivity and low specificity carries the risk of many false positives. In the present study, an optimal cutoff for predicting in-hospital mortality in NF patients appears to be a blood lactate level of 5.8 mmol/l, which has a sensitivity of 62% and a specificity of 86%. Our findings were similar to the results of a previous study conducted in patients with necrotizing soft tissue infection, where a lactate value of more than 6 mmol/l was a predictor of death [38]. Chang et.al found NF patients with hemorrhagic bullae, comorbidity with peripheral vascular disease, presence of bacteremia, or LRINEC score > 8 should receive early and primary amputation in order to prevent mortality [39]. Our data, together with previous data, suggest that NF patients with high blood lactate at ED should be closely monitored for signs of clinical deterioration and considered early intervention or primary amputation. Patients with low lactate levels should be considered for further monitoring of blood lactate, since serial lactate values may provide better prognostic information [21, 40–42].

The limitations of the present study include a temporal mismatch in evaluating mortality indicators. We compared the prognostic performance of a lactate value obtained on ED arrival to a SOFA score, which considers a range of values and includes the worst values obtained in the first 24 h of admission. The prognostic accuracy of the combination of blood lactate level and SOFA score was not significantly better than the use of SOFA score alone (*P* = 0.115), which might be explained by the discrepancy in the timescales for these two methods. Notably, the primary aim of the study was to evaluate the predictive value of blood lactate, when measured as a screening method at ED arrival, to predict mortality in patients with NF. Thus, serial changes in the lactate levels during the first 24 h of admission or post-surgical intervention were not evaluated. Trends in lactate concentration over time reflect the clinical response of patients to resuscitation and surgical intervention. Serial measurements of lactate levels might improve the sensitivity and specificity of this prognostic test [40–42]. Further studies are needed to investigate the trends in the changes of lactate values and explore whether the addition of the highest lactate value in the first 24 h to the SOFA score evaluation improves the prediction of mortality in NF patients.

Another limitation of the study is that during the blood collection period, a substantial number of patients might receive treatment with epinephrine under condition with bradycardia or shock. Epinephrine is known to affect lactate levels [43]. Unfortunately, we were unable to include these data in our multivariate analyses because we did not have information concerning therapeutic interventions for all NF patients.

## Conclusion

Our study indicates that the blood lactate levels upon ED arrival were significantly associated with mortality in NF, even after adjusting for age, serum albumin, and SOFA score. A high level of blood lactate upon ED arrival was independently predictive of in-hospital mortality in the NF patients. These findings extend the knowledge of blood lactate as a clinical biomarker of mortality in critical illness.

## Abbreviations

AUC: Area under the curve
CI: Confidence interval
CKD: Chronic kidney disease
CRP: C-reactive protein
DBP: Diastolic blood pressure
ED: Emergency department
LR+: Likelihood ratio positive
LR−: Likelihood ratio negative
NF: Necrotizing fasciitis
OR: Odds ratio
PV+: Positive predictive value
PV−: Negative predictive value
ROC: Receiver operating characteristic
SBP: Systolic blood pressure
SOFA: Sequential organ failure assessment
